# Supplementary material for: Inverse design of metal-organic frameworks using deep dreaming approaches
Source: Nat Commun. 2025 May 23;16:4806. doi: 10.1038/s41467-025-59952-3 (PMC12102185; doi:10.1038/s41467-025-59952-3)
Supplement: Supplementary file 1 — Supplementary Information [file 41467_2025_59952_MOESM1_ESM.pdf]

Supporting Information: Inverse design of metal-organic frameworks using deep dreaming approaches

Conor Cleeton* and Lev Sarkisov

Department of Chemical Engineering, University of Manchester, Manchester

E-mail: conor.cleeton@manchester.ac.uk

Contents

Supplementary Note 1: Group SELFIES representation of edge SBUs **3**

Supplementary Note 2: MOF string vocabulary **6**

Supplementary Note 3: Deep dreaming regression performance plots **10**

Supplementary Note 4: Linker validity constraints **11**

Supplementary Note 5: Shifting property distributions **14**

Impact of structural relaxation 15

Supplementary Note 6: Automated interpretation of transmutation pathways **18**

Supplementary Note 7: Model uncertainty quantification **22**

Supplementary Note 8: Multiobjective optimisation of uncorrelated properties **24**

Supplementary Note 9: Deep dreaming compared to other generative modelling approaches **25**

Supplementary Note 10: Comparing linker string representations **36**

Supplementary Note 1: Group SELFIES representation of edge SBUs

In this work, we use Group SELFIES[1] strings to represent edge SBUs. In addition to the semantically robust alphabet of SELFIES,[2] we encode common substructures of known MOF materials using Group tokens, as shown in Figure S1. These Group tokens were chosen to be consistent with the functional groups used to augment / expand the diverse edge dataset

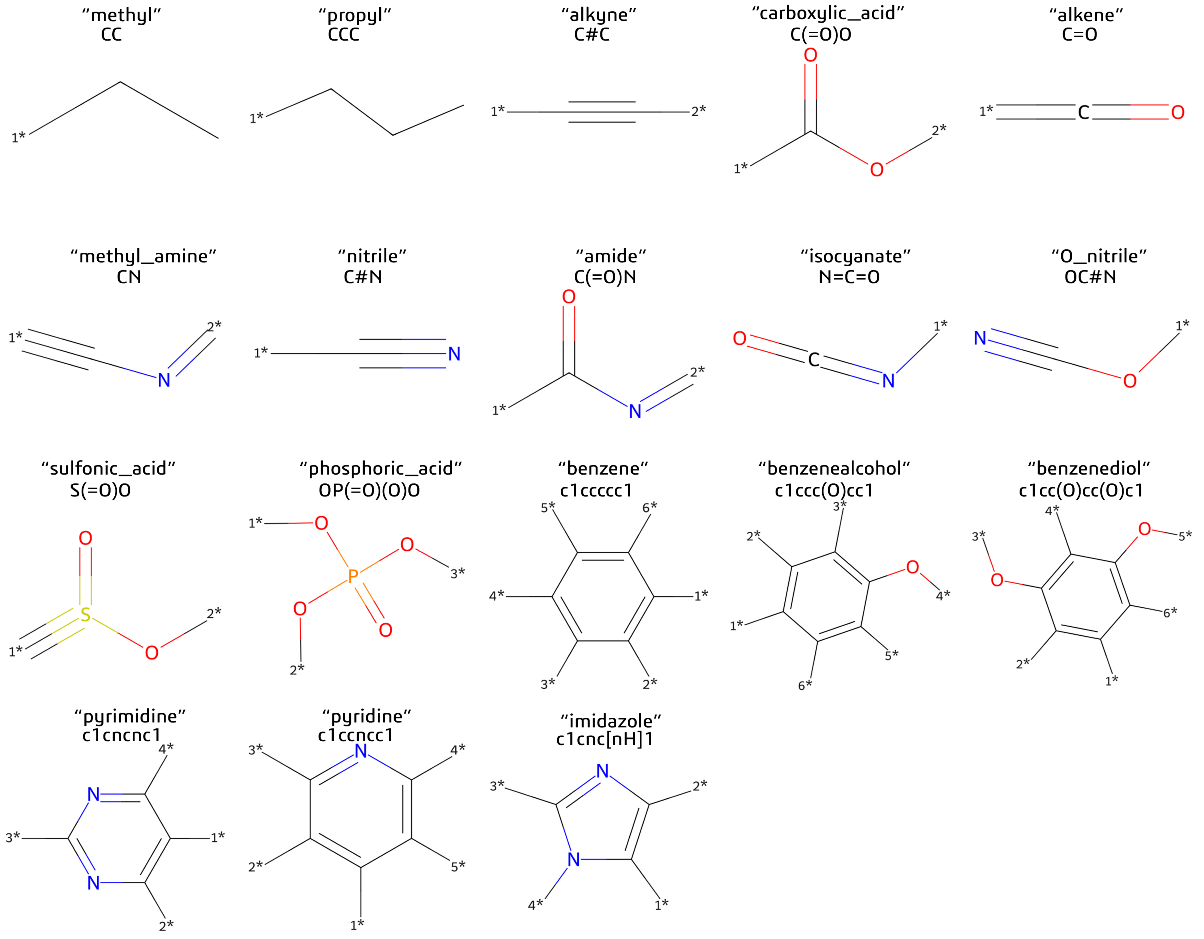

Figure S1: **Group SELFIES tokens used to encode MOF edge SBUs**. The Group SELFIES token is provided in quotation marks above each substructure, along with the relevant SMILES string. The *N notation represents the Nth attachment point, indicating how the group can participate in bonding.

of Yao et al.,[3] which is the dataset of linkers we sampled from to train our deep dreaming models (see the *Dataset curation* section in the main article). Note that functional groups such as F, Cl, Br, and OH are represented by single SELFIES tokens ([F], [Cl], [Br], [O]) and thus do not require explicit Group tokens.

The decision to use Group SELFIES for the edge SBU was chiefly motivated by the fact that relevant chemical substructures from the MOF seed (the initial structure to be optimised) are rarely preserved in deep dreaming experiments when SELFIES[2] strings are used. For example, in Figure S2 we show how simple operations such as token substitution, addition / subtraction, or shuffling, affects the molecular character of the initial seed. Important scaffolds such as the aromatic rings are often lost after these operations are executed on the SELFIES representation. Conversely, because pyridine and benzene are explicitly encoded using the `[:2pyridine]` and `[:0benzene]` Group SELFIES tokens, respectively,

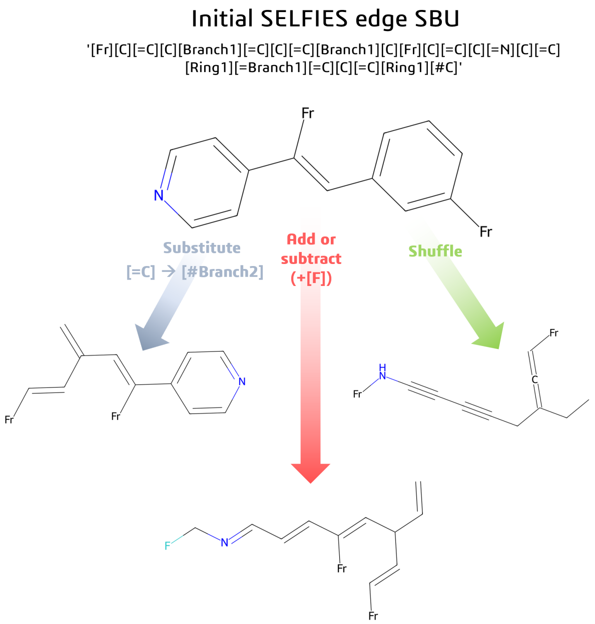

Figure S2: **SELFIES vs Group SELFIES representation of edge SBUs**. Taking the same edge SBU as the initial seed structure, we encode both the SELFIES and Group SELFIES representations. Three separate operations are performed on the seed encodings: (1) a single token is substituted with another token randomly (blue arrow); (2) a single token is randomly inserted or deleted (red arrow); (3) the original encoding is randomly shuffled (green arrow). Fr is a pseudoatom used to represent the connection points of the linker.

they are more frequently observed in the decoded structures. This simple example is used as a proxy demonstration of the deep dreaming experiment, where seed structures are subject to multiple token substitutions, additions, and subtractions during optimisation.

Supplementary Note 2: MOF string vocabulary

Table S1: Edge SBU Group SELFIES token vocabulary.

[nop]	[#Branch]	[#C]	[#N]
[->]	[#:1methyl_amine]	[:00_nitrile]	[:0alkene]
[:0amide]	[:0benzene]	[:0benzenealcohol]	[:0benzenediol]
[:0carboxylic_acid]	[:0imidazole]	[:0isocyanate]	[:0methyl]
[:0methyl_amine]	[:0nitrile]	[:0phosphoric_acid]	[:0propyl]
[:0pyridine]	[:0pyrimidine]	[:0sulfonic_acid]	[:1alkyne]
[:1amide]	[:1benzenealcohol]	[:1benzenediol]	[:1carboxylic_acid]
[:1imidazole]	[:1methyl_amine]	[:1pyridine]	[:1pyrimidine]
[:1sulfonic_acid]	[:2benzenealcohol]	[:2benzenediol]	[:2imidazole]
[:2pyridine]	[:2pyrimidine]	[:3benzenealcohol]	[:3benzenediol]
[:3imidazole]	[:3pyridine]	[:3pyrimidine]	[:4benzenealcohol]
[:4benzenediol]	[:4pyridine]	[:5benzenealcohol]	[:5benzenediol]
[:=0amide]	[:=0methyl_amine]	[:=1methyl_amine]	[=Branch]
[=C]	[=N]	[=O]	[=P]
[=Ring1]	[=Ring2]	[=S]	[Br]
[Branch]	[C]	[Cl]	[F]
[FrH0]	[N]	[O]	[P]
[Ring1]	[Ring2]	[SH2]	[S]
[pop]			

Table S2: Pormake nodes used to construct the training set.

N100	N101	N105	N108	N109	N112	N115	N117
N125	N127	N128	N133	N136	N137	N139	N143
N144	N146	N147	N148	N151	N152	N153	N156
N158	N161	N162	N164	N167	N17	N170	N173
N174	N178	N180	N183	N184	N186	N188	N192
N193	N194	N2	N200	N205	N206	N209	N211
N213	N214	N219	N220	N222	N233	N234	N235
N238	N240	N245	N246	N250	N254	N256	N257
N259	N262	N263	N265	N267	N27	N274	N282
N283	N284	N288	N29	N292	N295	N298	N302
N304	N307	N308	N31	N310	N314	N315	N317
N32	N321	N324	N328	N330	N332	N335	N336
N337	N34	N341	N342	N343	N346	N348	N35
N350	N355	N359	N36	N360	N364	N365	N366
N37	N371	N372	N375	N376	N379	N381	N383
N390	N392	N393	N398	N409	N411	N415	N417
N42	N424	N425	N432	N434	N435	N437	N440
N445	N447	N448	N45	N450	N455	N461	N464
N47	N470	N472	N480	N483	N485	N486	N489
N491	N492	N493	N494	N50	N500	N503	N505
N507	N508	N51	N512	N513	N515	N519	N520
N521	N524	N525	N526	N529	N533	N534	N538
N539	N540	N543	N545	N547	N549	N55	N551
N553	N555	N556	N557	N559	N560	N561	N569
N572	N574	N579	N580	N581	N583	N585	N591
N593	N595	N598	N60	N601	N606	N611	N613
N618	N621	N624	N627	N628	N641	N645	N65
N654	N657	N666	N668	N678	N68	N680	N681
N682	N689	N691	N694	N696	N702	N708	N709
N713	N715	N717	N718	N719	N76	N85	N87
N89	N90	N93	N96				

Table S3: Node SBU SELFIES token vocabulary.

[nop]	[#Branch1]	[#Branch2]	[#C]	[&&]	[=Branch1]
[=Branch2]	[=C]	[=N]	[=O]	[=P]	[=Ring1]
[=Ring2]	[=S]	[Ag]	[Al]	[B]	[Be]
[Bi]	[Br]	[Branch1]	[Branch2]	[C]	[Cd]
[Ce]	[Cl]	[Co]	[Cr]	[Cu]	[Dy]
[Er]	[Eu]	[F]	[Fe]	[Fr]	[Gd]
[Ge]	[Ho]	[I]	[In]	[La]	[Lu]
[Mg]	[Mn]	[Mo]	[N]	[Nd]	[Ni]
[O]	[P]	[Pd]	[Pr]	[Pt]	[Rh]
[Ring1]	[Ring2]	[S]	[Sc]	[Si]	[Sm]
[Tb]	[Tm]	[U]	[Y]	[Yb]	[Zn]

Table S4: Topology (tokens) which are used to construct MOFs in pormake.

[acs]	[ana]	[bcg]	[bcu]	[bnn]	[cag]
[cds]	[crb]	[crs]	[dia-f]	[dia]	[dmp]
[ecu]	[eta]	[gis]	[gme]	[hex]	[lcy]
[lon]	[lvt-a]	[lvt]	[nbo-a]	[nbo]	[neb]
[pcu]	[qtz]	[reo]	[rho]	[rob]	[sod]
[sra]	[srs]	[sxb]	[ths]	[unc]	[utk]
[utp]					

Supplementary Note 3: Deep dreaming regression performance plots

We report the regression performance of our model, trained using 10,000 data points, in Table S5. Evidently, the deep dreaming architecture is capable of predicting out-of-sample testing data with good accuracy for a diverse set of material properties. Almost perfect agreement is observed between the reference and predicted c_p values. Despite the fact that no 3D coordinate information is encoded into the MOF strings, the model shows respectable accuracy in predicting the GSA and VF. For the bandgap, Q_{CO_2}, and S_{CO_2/N_2}, the agreement between "ground truth" data and the model predictions are more modest. Learning curves for these properties indicate that adding more training data would likely lead to better performance. However, we found 10,000 training points to be acceptable for the purposes of our investigation. Parity plots of the model predictions are provided in Figure S3.

Table S5: Regression metrics for the predictive performance of the deep dreaming model for several MOF properties.[a] R^2 is the coefficient of determination, SRCC is the Spearman ranking correlation coefficient, and MAE is the mean absolute error.

	GSA (m^2 g^{-1})	VF (-)	c_p (J g^{-1} K^{-1})	Bandgap (eV)	Q_{CO_2} (kJ mol^{-1})	S_{CO_2/N_2} (-)
R^2	0.93	0.91	0.99	0.71	0.76	0.69
SRCC	0.96	0.95	0.99	0.83	0.90	0.85
MAE	368.45	0.05	0.01	0.34	4.14	2.24

[a]Note that a separate model is trained for each property.

Supplementary Note 4: Linker validity constraints

By design, all molecules generated during deep dreaming are chemically valid, however they may not necessarily be valid from a linker chemistry point of view. Two connection points are required to form bonds with the node SBUs in a given topology. To facilitate the formation of these connection points, we include an additional loss term to the target losses (computed as the mean squared target error) during the deep dreaming process. As each token position is represented by a probability distribution across the edge SBU vocabulary, we can represent the "probability mass" of each token as the sum of their continuous, noisy one-hot activations in each token position, which we will denote p_{token}. We focus on the token representing a connection site — labelled as `[FrH0]` in our vocabulary — such that the p_{Fr} allocated to this token across the sequence corresponds to the effective number of connection points. Rather than enforcing a hard constraint by decoding to a discrete molecule and counting the exact number of francium pseudoatoms, we introduce a smooth penalty based on a "soft count" of `[FrH0]`. Specifically, we apply a quadratic penalty, $\alpha(p_{Fr}-2)^2$, to discourage deviations from the desired count of two connection points. Because p_{Fr} remains a real-valued, differentiable function of the underlying continuous representation, the gradient is well-defined even when p_{Fr} is near 2. This preserves a smooth gradient signal throughout deep dreaming, while still yielding valid, integer-valued molecular compositions upon decoding.

It is also important that these connection points do not arise too close together to avoid the formation of unphysical structures. To inform our decision on what constitutes a physically meaningful distance between connection points, we consider the edge dataset used to train our model, which was obtained from Yao et al.[3] Yao et al. randomly functionalised linkers extracted from the CoRE MOF database,[4] which are a subset of MOFs that have been experimentally realised and thus represent linkers with physically meaningful connection point distances. We therefore compute the distance between connection points based on graph connectivity for these linkers and normalise them relative to the maximum distance

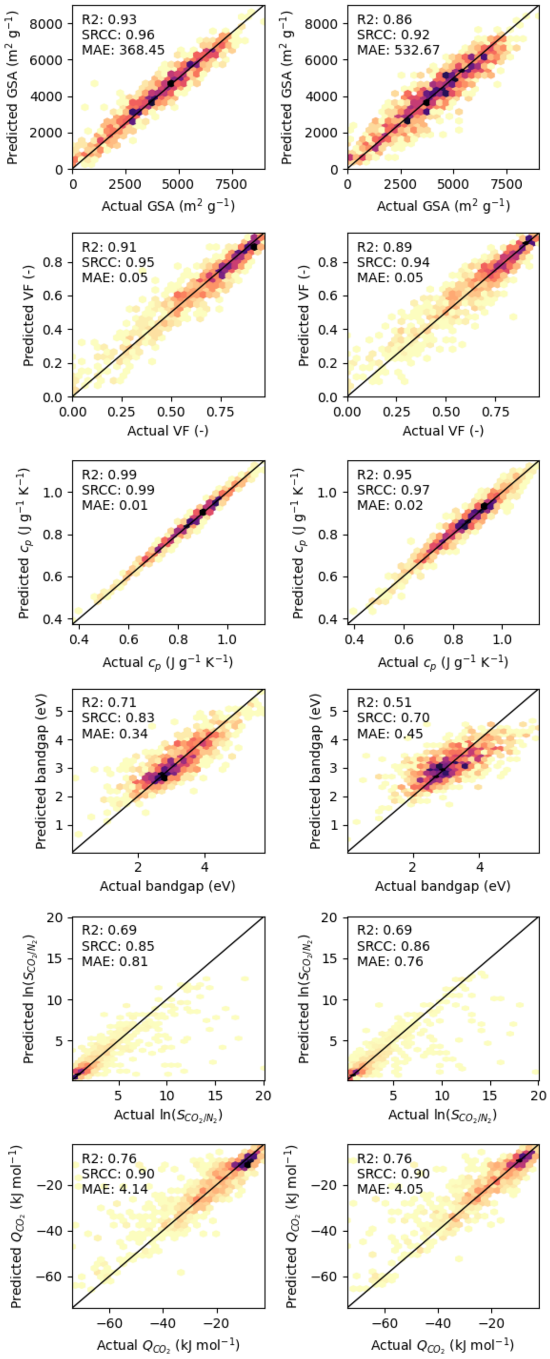

Figure S3: **Parity plots of the deep dreaming model out-of-sample predictive performance for several MOF properties**. Hexagonal binning is used to represent the density of points, with dark colours representing a high density and light colours representing a low density. The top row shows the models trained on the noise-free MOF strings, while the bottom row shows the performance of the models trained on noisy (k = 0.6) representations. Regression metrics, such as the coefficient of determination (R2), Spearman rank correlation coefficient (SRCC), and mean absolute error (MAE), are displayed in each subplot. The line of parity is given by the solid diagonal line. Note that 1,000 out-of-sample data points are used to generate these figures and derive the relevant statistics. Source data are provided as a Source Data file.

path of the molecule. Most of the linkers are formed by connection points with normalised distances ≥ 0.6, as shown in Figure S4. We assign this as our constraint for linker validity.

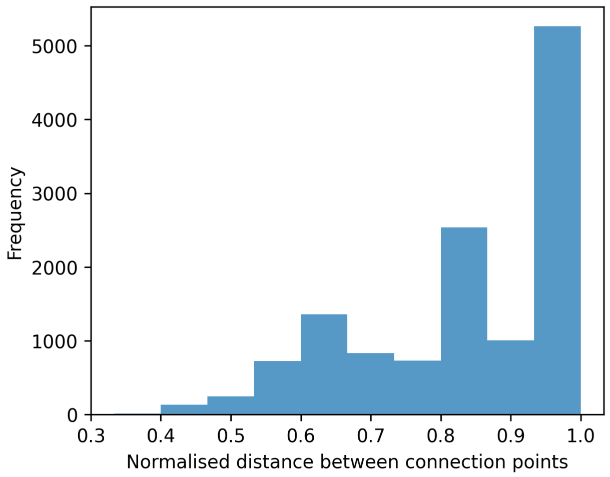

Figure S4: Normalised graph distances between connection points in linkers obtained from the dataset of Yao et al.[3] Source data are provided as a Source Data file.

Supplementary Note 5: Shifting property distributions

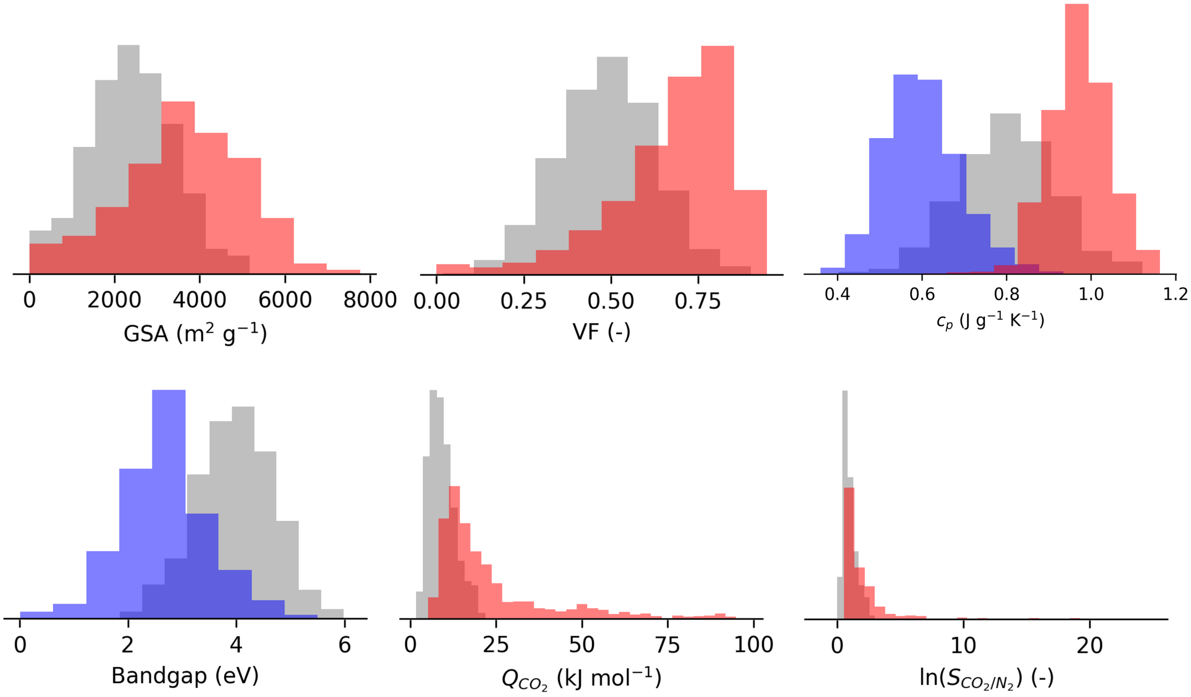

Figure S5: **Single objective optimisation of several MOF properties.** Here we show the optimisation results for 1,000 seed MOFs in each case study. Blue distributions refer to property values that are minimised, while red distributions refer to property values that are maximised. Typically, we wish to maximise GSA and VF to improve the adsorption properties of MOFs. The c_p of MOFs may be minimised or maximised, depending on the target application. Low bandgap MOFs are desirable for the next generation of semiconductors, and so this property value is minimised. In the context of direct air capture applications, we wish maximise the Q_{CO_2} and S_{CO_2/N_2}. Note that the "ground truth" values are shown in each of these distributions, recomputed using either high-fidelity simulation or via machine learning surrogates (see *MOF property determination* in the Methods section in the main article). Source data are provided as a Source Data file.

Impact of structural relaxation

Given the hypothetical nature of the generated MOFs, their crystalline structures are unlikely to be perfect. Therefore, structural relaxation is generally required to optimise their atomic coordinates and achieve more realistic configurations. Although the results in the main article are derived from unrelaxed structures to initially showcase the deep dreaming approach for inverse MOF design, incorporating this additional post-processing step will likely be required before translating hypothetical MOFs into real materials.

To evaluate the impact of this optimisation on MOF properties, we selected a representative subset of $n = 100$ MOFs from the distributions presented in Figure S5 (of size $n =$ 1,000). We fitted a probability density function to these distributions and drew samples that retained the features of the original datasets. These MOFs were then structurally optimised using the UFF forcefield as implemented in the Forcite Module of Materials Studio 2019,[5] with property values recalculated as outlined in the *MOF property determination* section of the main article.

In the case studies for optimising c_p, our findings reveal that structural relaxation has a minimal impact on the overall results. The mean absolute error (MAE) between the relaxed and unrelaxed structures is approximately 0.03 J g^{-1} K^{-1}, indicating a deviation of around 3.75%. This comparison is detailed in Figure S6. However, more significant differences are observed in other parameters, including the bandgap, Q_{CO_2}, S_{CO_2/N_2}, and gravitational surface area (GSA). The respective MAEs for these parameters are about 0.64 eV, 5.94 kJ mol^{-1}, 0.79, and 288.5 m^2 g^{-1}, translating to average deviations ranging from 15% to 30%. These differences are comparable to the prediction errors of our ML models, as reported in Table S5. Most notably, the MAE for pore volume (VF), as shown in Figure S6, is 0.27, which is approximately five times greater than the prediction errors in Table S5.

Evidently, structural relaxation plays a critical role in accurately evaluating properties that solely rely on the exact atomic coordinates (i.e., VF), an important role for other properties that depend on both the chemistry and structural features (i.e., the bandgap,

Q_{CO_2}, and S_{CO_2/N_2}), and a minimal role for properties predominantly determined by the material's chemistry, such as the c_p.

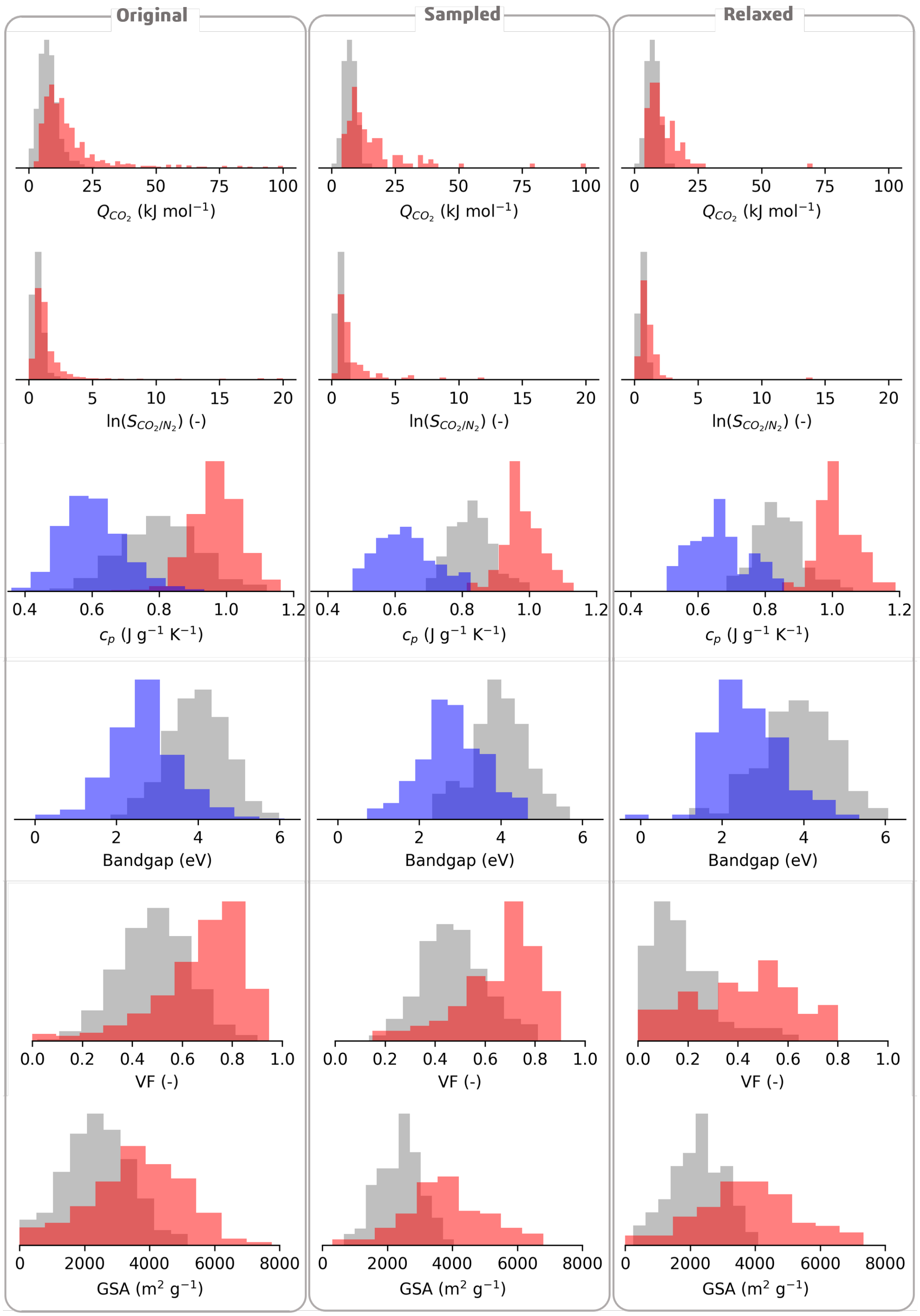

Figure S6: **Impact of structural optimisation on MOF property distributions.** We strategically sampled 100 MOFs (middle column) from the original dreamed distributions of size 1,000 (left column), which are obtained from unrelaxed structures. We show how these sampled distributions are affected by structural relaxation in the right column. Source data are provided as a Source Data file.

Supplementary Note 6: Automated interpretation of transmutation pathways

To help uncover important design rules, we have provided functionality that automates the interpretation and visualisation of deep dreaming pathways. This is demonstrated in a case study focussed on minimising the c_p of a MOF.

In Figure S7, we show how, in charting a path towards a MOF with lower c_p, the model opts to retain the pyridine substructure of the seed linker while appending bromine atoms to the molecular backbone. This is noted as a consistent strategy in this particular optimisation, where the model identifies the important tokens utilised at steps between *valid* linker transmutations. The intermediate steps are the collection of modifications suggested by the model that are invalid, according to our definition of linker validity in **Supplementary Note 4**. In particular, `[Br]` tokens and nitrogen-containing Group tokens (such as `[pyrimidine]` and `[pyridine]`) are considered important in these intermediate pathways.

Importantly, different pathways may emerge from the same seed structure under different noise initialisations. As shown in Figure S8, this pathway favours sulphur and chlorine additions, while another pathway (in Figure S9) prefers the addition of sulphur followed by bromine. These visualisations illustrate how the same optimisation objective can be achieved through multiple distinct routes.

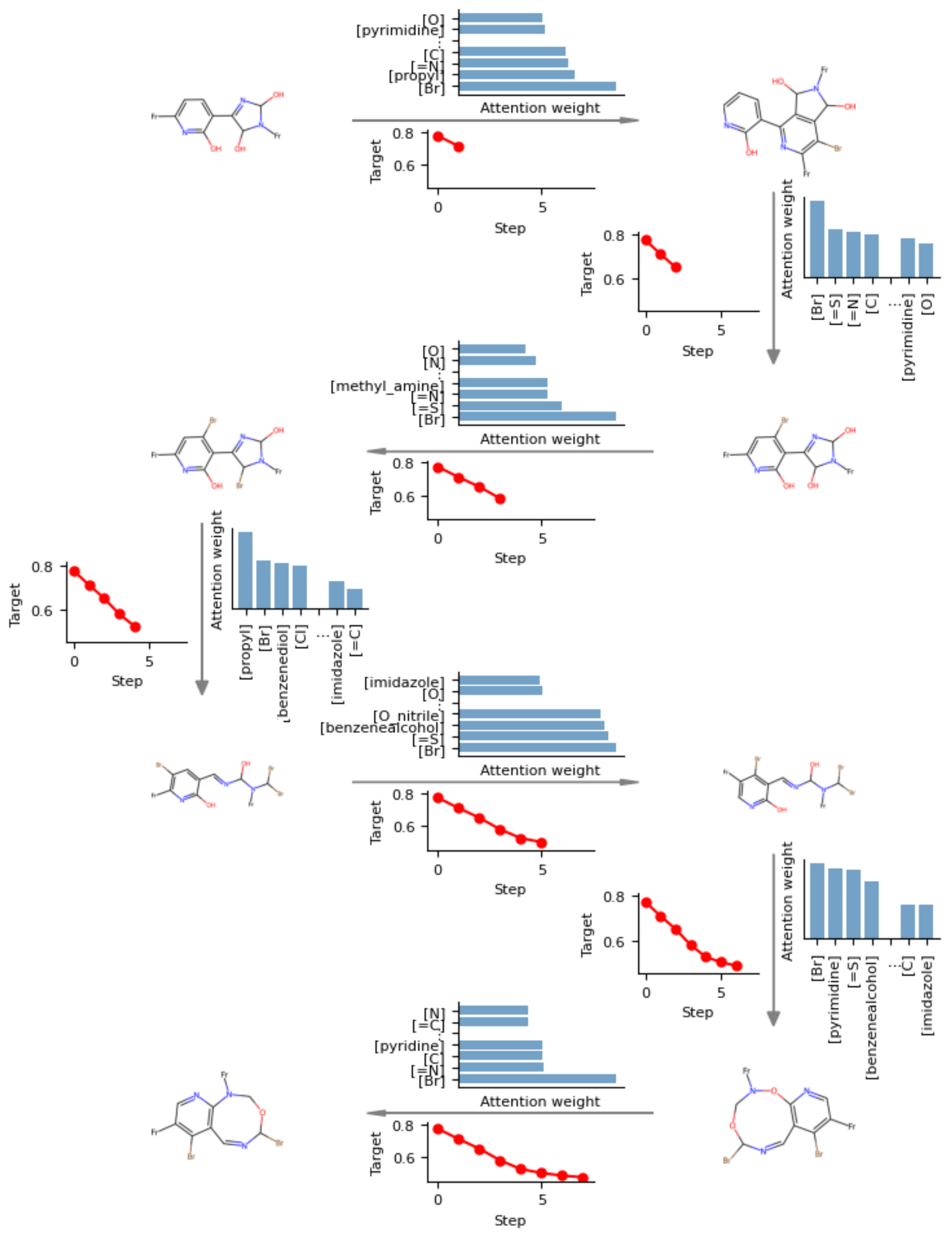

Figure S7: This figure illustrates the valid transmutation pathway in a snake-like pattern, from left to right, starting from the seed linker to the final optimised linker. At each step, we compute normalised attention score distributions for the tokens of molecules suggested by the model to traverse one valid transmutation to the next. These scores are used to evaluate the importance of each token, with the median values plotted in descending order to highlight their relative importance in the intermediate pathways. Additionally, the target property value is recorded at each valid step, demonstrating how it changes in response to the transmutations. In this example, the bromine token consistently ranks among the top two most important tokens, reflecting its critical role in the desired objective of minimising the c_p of the seed MOF.

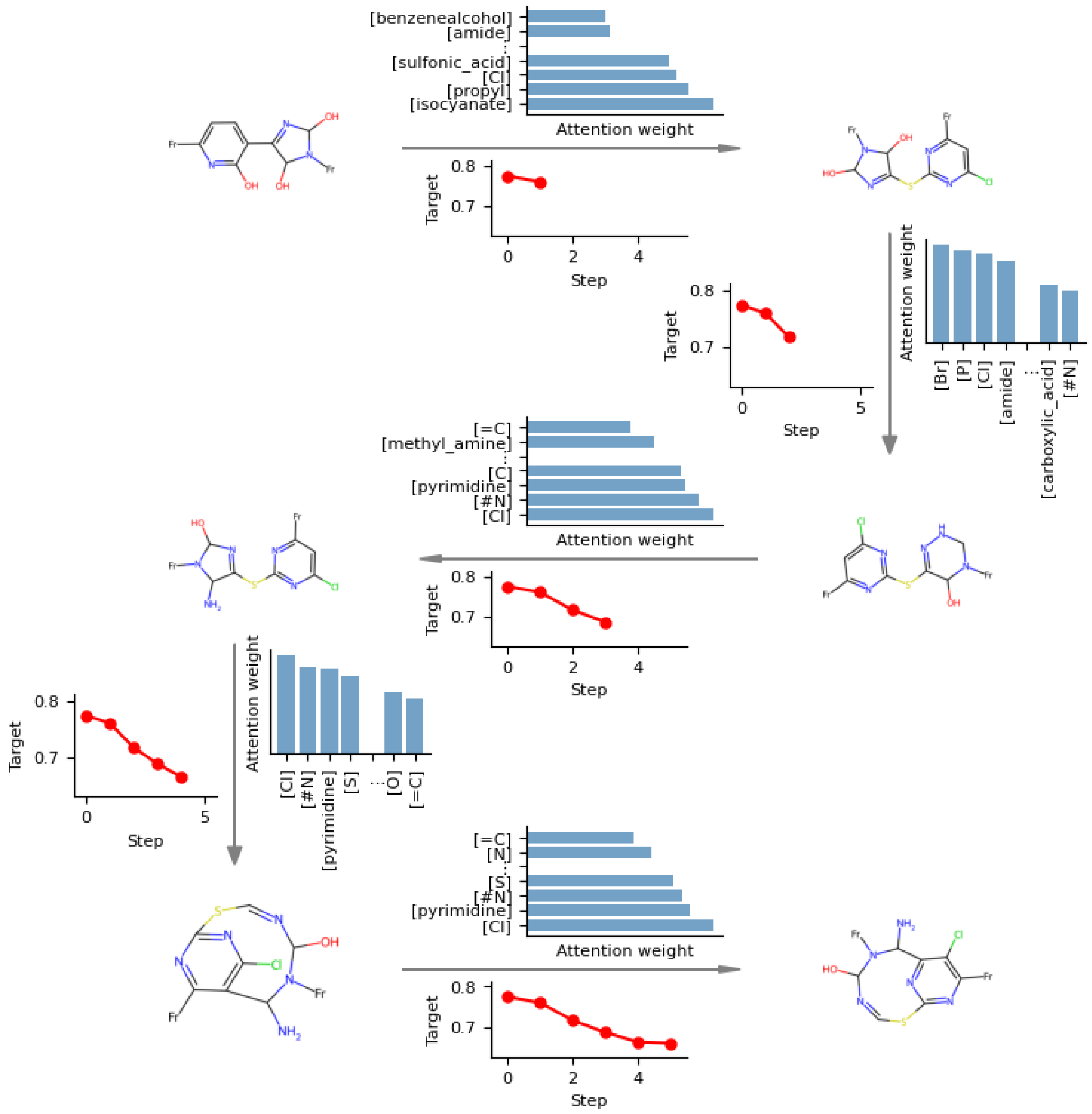

Figure S8: This figure illustrates an example whereby, in order to minimise the c_p of a seed MOF, the model charts a pathway that considers sulphur and chlorine tokens as important tokens in the first few transmutations, followed by the pyrimidine group token, chlorine, and sulphur SELFIES tokens in the last few transmutations. The importance of these tokens manifest in the decoded structures, whereby we see sulphur-, chlorine-, and nitrogen-containing groups as a common strategy in the optimisation pathway.

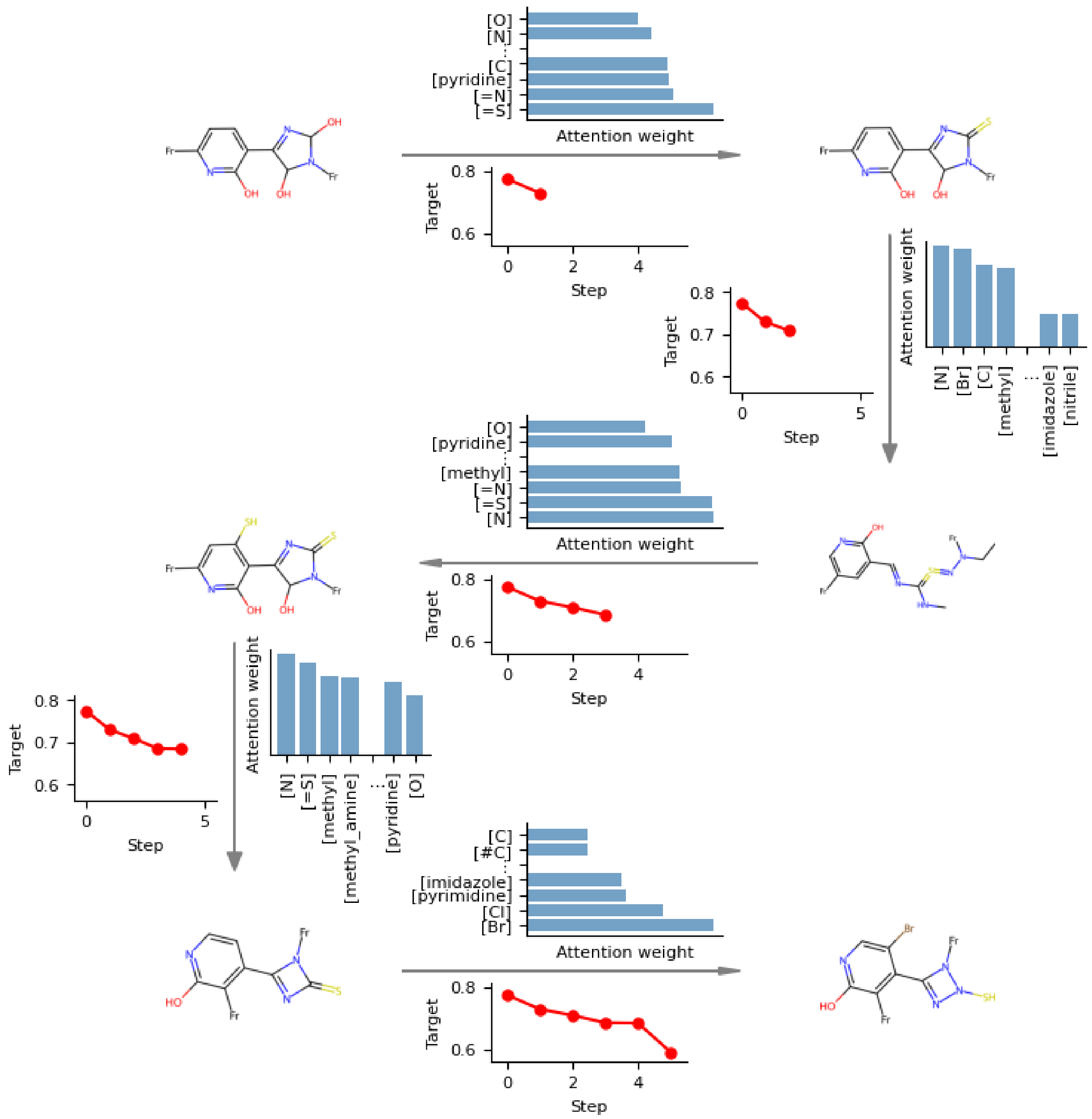

Figure S9: This figure illustrates an example whereby, in order to minimise the c_p of a seed MOF, the model charts a pathway that considers sulphur- and nitrogen-containing tokens as important transmutations in the first instance, followed by the importance of bromine tokens in the last transmutation. As in the previous case studies, the importance of these tokens manifest in the valid decoded molecules.

Supplementary Note 7: Model uncertainty quantification

Deep dreaming relies on using a regression model to reverse engineer new MOFs. These architectures are typically designed to interpolate within the range of data they have been exposed to during training. When tasked with predicting properties or generating structures that are outside this range, there will invariably be some challenges with extrapolation.[6]

To illustrate the novelty of the linkers optimised for the c_p case study in the main article (*Shifting property distributions* section), we employed a t-SNE projection to visualise the chemical space coverage relative to the training set. This revealed that our dreamed linkers can map to points far from the training manifold. Here, we group MOFs in the dreamed distributions based on the Tanimoto similarity of the linker to their nearest neighbour linker in the training set and computed the average prediction errors within each group. Our findings, depicted in Figure S10, indicate that MOFs with lower Tanimoto similarity (i.e., with features more dissimilar to the training set) exhibit greater prediction errors compared to those with higher similarity. This observation highlights the regions where the model's predictions are less reliable.

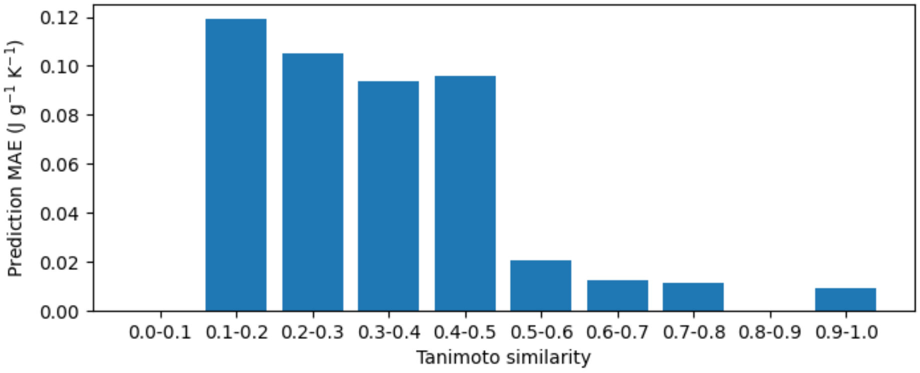

Figure S10: Prediction mean absolute error (MAE) for c_p-optimised MOFs, grouped according to the Tanimoto similarity of their dreamed linkers relative to their nearest neighbour linker in the training dataset. MOFs with lower Tanimoto similarities show higher prediction MAEs. We use $\sim$ 2,000 data points to generate this figure. Source data are provided as a Source Data file.

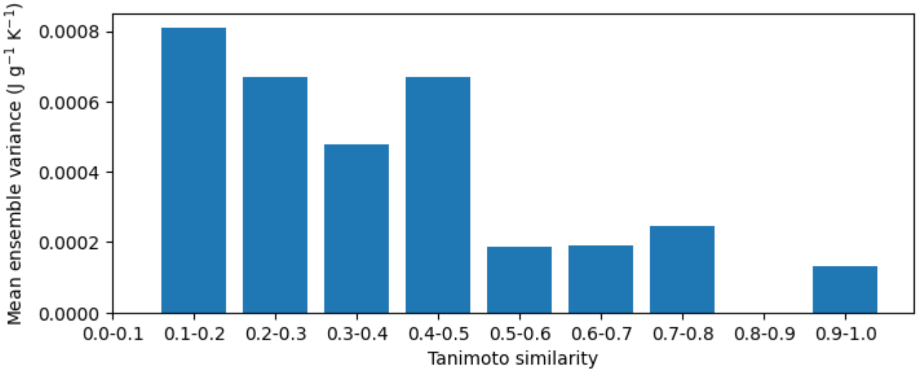

Figure S11: The uncertainty in the deep dreaming model predictions, grouped according to the Tanimoto similarity of their dreamed linkers relative to their nearest neighbour linker in the training dataset. We use $\sim$ 2,000 data points to create this figure. Source data are provided as a Source Data file.

To address the issue of generalisation, we can adopt "uncertainty-aware" deep dreaming models by implementing an uncertainty quantification approach. For example, we train 10 deep dreaming models using different training sets, resampled with replacement, and estimate the uncertainty in our model using the variance in the predictions of the ensemble. By grouping these uncertainty estimates by Tanimoto similarities, similar to Figure S10, we find that this method is fairly effective at differentiating between regions of high and low uncertainty (Figure S11). This additional layer of uncertainty quantification provides valuable insights into the model's reliability across various chemical spaces. It also supports a more informed exploration of novel chemical spaces through an iterative generative and training feedback loop. Specifically, samples identified with high uncertainty could be prioritised for inclusion in the training set in subsequent model iterations, enhancing the model's generalisability. By incorporating these strategies, practitioners may expand the applicability beyond the initial training domain, which, in turn, can facilitate more reliable MOF design.

Supplementary Note 8: Multiobjective optimisation of uncorrelated properties

In addition to an appreciable heat of CO_2 adsorption at infinite dilution, Q_{CO_2}, MOFs with low gravimetric heat capacities, c_p, are desired for direct air capture (DAC) applications. Therefore, as a supplement to the multiobjective case study of Q_{CO_2} and S_{CO_2/N_2} in the main article, we seek here to minimise c_p and maximise Q_{CO_2} simultaneously. This optimisation problem is more challenging as c_p and Q_{CO_2} are relatively uncorrelated (i.e., a Pearson correlation of 0.24 is computed from the training data) compared to Q_{CO_2} and S_{CO_2/N_2} (with a Pearson correlation of 0.94). In Figure S12, we show the dreamed distributions obtained after optimising 100 MOFs. We observe similar shifts in the multiobjective property distributions compared to the single objective case studies shown in Figure S5. Over 80% of the dreamed MOFs show improved material properties for both c_p and Q_{CO_2} compared to their seed structures, demonstrating that both properties are indeed being optimised simultaneously.

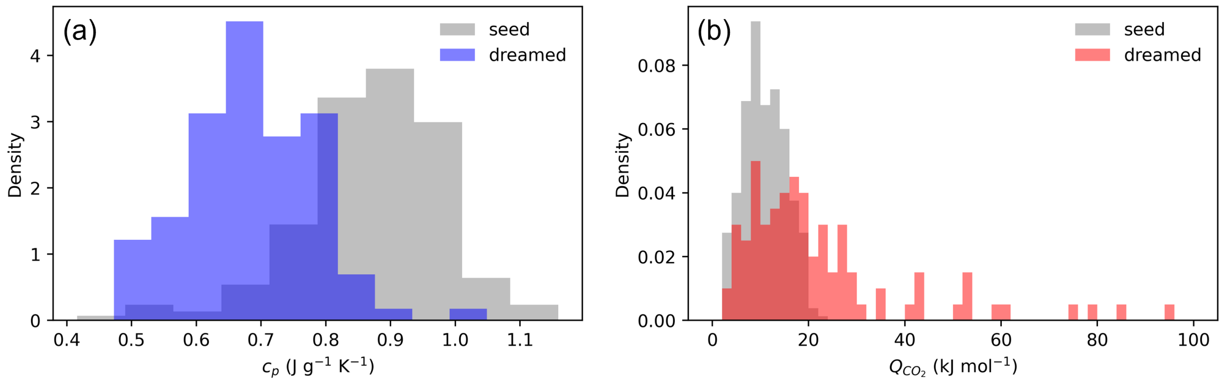

Figure S12: **Multiobjective optimisation of c_p and Q_{CO_2}.** From a seed distribution of 100 MOFs, (a) and (b) show the dreamed distributions obtained from minimising c_p and maximising Q_{CO_2} simultaneously. Note that these distributions show the recomputed "ground truth" values obtained from the ML model of Moosavi et al.[7] (for c_p) and molecular simulation (for Q_{CO_2}) using unrelaxed crystal structures. Source data are provided as a Source Data file.

Supplementary Note 9: Deep dreaming compared to other generative modelling approaches

Given the complexity of reticular materials relative to other, simpler molecules and materials (such as small inorganic crystals), inverse design of complete MOF structures at atomic resolution has not yet been achieved. This arises for several reasons: (1) organic-inorganic complexes like MOFs are comprised by diverse element types compared to small organic molecules, which usually only contain C, N, O, and H; (2) direct optimisation of 3D crystal structures has been rare due to the challenges in obtaining a continuous representation; (3) MOF unit cells are much larger than small inorganic crystals (on the order of 10^2–10^3 as opposed to 10^1); and (4) there are complex chemistry constraints associated with metal clusters that typically preclude the use of atomic-scale generative modelling. It is therefore common to abstract the representation of MOFs to the level of structural building units (SBUs), and deploy inverse design principles towards optimising these SBUs and their connectivity (i.e., the topology).

Focussing on this coarse-grained representation of MOFs, there are two classes of approaches to inverse design worth differentiating. The first class belongs to what we call "SBU library" methods: these seek to find the optimal combination of SBUs and topologies from a library of predetermined building blocks and topological nets. This may be achieved with the use of genetic algorithms,[8] GFlowNets,[9] and diffusion models,[10,11] for example. The other class of generative models, which our MOF dreaming approach belongs to, are those that seek to generate novel (linker) building blocks. This may be the sole focus of the inverse design, as in our study, or it may be complimented by an additional search for optimal node SBUs and topological nets, as in other studies. The relevant works in this context are by Yao et al.[3] who developed a generative model based on the principles of variational autoencoders, Park et al.[12] who utilise diffusion models and regression models for MOF design, and Park et al.[13] who use transformers and reinforcement learning.

While the objective of inverse design remains consistent, a systematic benchmarking effort with precedent models is not possible, currently. This limitation stems partly from the diversity in the datasets used for training, the design focus, optimisation strategies, and the target KPIs, which can vary widely. More critically, the quality of samples produced by generative models remains unquantifiable, since a clear definition of "quality" for MOFs has yet to be established. For instance, as we explore in the main article, practical aspects like the synthesisability of MOFs still lacks a clear framework for thorough evaluation within the MOF community. We must therefore be cautious in our selection of parameters and models when making comparisons.

Here, we compare in more detail the differences between our MOF dreaming model and generative models that belong to the second class of inverse design approaches. Although a thorough assessment of sample quality is currently unfeasible, we can still make meaningful quantitative comparisons based on criteria such as the training requirements (number of

Table S6: Deep dreaming compared to other generative modelling approaches for MOF design.

	Deep dream (this work)	Sm-VAE[3]	GHP-MOFassemble[12]	RL transformer[13]
Training requirements	12K labelled	45K labelled<br>~2M unlabelled	~300K unlabelled organic molecules<br>~137K labelled MOF structures	24-33K labelled<br>~1.9M unlabelled
Design focus	Linker	Full MOF	Linker	Full MOF
Unconditional or targeted generation?	Targeted generation only	Both	Unconditional generation only	Both
Optimisation approach:<br>• scope<br>• strategy<br>• chemical space	• local<br>• gradient-based<br>• real	• local<br>• gradient-based<br>• latent	N/A[a]	• global<br>• reinforcement learning<br>• real
Validity (%)	95.5	61.5	15.6	69
Uniqueness (%)	99.4	99.7	67.5	79

[a] There is no direct optimisation of structures in this approach. The authors generate linkers via unconditional diffusion, construct MOFs with these linkers, and then screen for a desired material property afterwards.

labelled and unlabelled data points) and the validity / uniqueness of the structures generated. Importantly, a structure that is "valid" does not necessarily mean "realistic", and we discuss more on what constitutes a "realistic" structure in the main article. However, the "validity" of MOFs generated through inverse design typically refers to whether the correct reticular information could be sampled, i.e., its CIF file could successfully be constructed and, in some cases, whether the 3D atomic coordinates of the MOF could be optimised through structural relaxation. "Uniqueness" is often related to how many non-duplicate organic linkers are produced in the generative process. Where possible, we make comparisons between models on a consistent basis in these coordinates in Table S6, and reflect on the qualitative differences between each approach below.

Deep dreaming (this work)

- **Summary:** *Benefits:* Deep dreaming requires the lowest training requirements of all the models considered in Table S6. MOFs are also generated with the highest validity. Direct optimisation of these linkers occurs within the real chemical space, which helps reveal what the model has learned of the structure-property relationship. Our model is also useful because many researchers develop machine learning models for property predictions. By leveraging our approach, users can create generative strategies on top of existing models, provided that the MOFs are encoded using molecular strings. This capability enhances the utility of current predictive models by enabling the generation of new MOF structures that meet specific design criteria. *Drawbacks:* its design focus is limited to MOF linkers, and the algorithm used to optimise these linkers relies on gradient-descent, which is local in scope.

- **Overview:** The model architecture is described in full detail in the main article.

- **Training requirements:** Relative to other generative approaches, our method is relatively "light-weight", in that only 12,000 labelled MOF structures were used to

achieve good regression performance (Figure S3). As the same architecture is used for the task of inverse design, the full training requirements are embodied by the dataset needed for the regression task. Our training set was generated using *pormake*, as described in the methodology of the main article.

- **Design focus:** Metal clusters are not amicable to continuous differentiation in the same way that MOF linkers are. For this reason, our design focus has been on linker design, while keeping the node and topology fixed. By focussing only on the linker representation — which is encoded using a 100% robust molecular string and is optimised to always guarantee a valid linker — we are able to design MOFs with very high success rate (∼96% validity, see *Validity* discussion below). Nevertheless, this benefit is counterbalanced by its more limited (local) scope, which we discuss more about below.

- **Unconditional or targeted generation:** Deep dreaming differs from typical generative models. Our approach does not learn to unconditionally generate samples that follow a training distribution like, for example, the Sm-VAE or RL transformer models. Our model only performs conditional (targeted) generation, as we learn a mapping between structures and their properties first ($f(X) \rightarrow \hat{y}$) and then invert the architecture to obtain new MOFs ($f(\hat{y}) \rightarrow X$) with improved property values. To do this, we must always define a value $\hat{y}$ and optimise X towards this value.

- **Optimisation approach:** As described in the main article, we use gradient-based algorithms to optimise the linker representations, which are local in scope. This optimisation does, however, occur in the real chemical space rather than within a latent space, which is a notable advantage of deep dreaming due to its inherent interpretability.

- **Validity:** In our work, we define a MOF as valid if: (1) its 3D coordinate information is successfully generated and saved in CIF format from its optimised MOF string

representation, and (2) the CIF file successfully undergoes structural relaxation. We report a validity of 95.5% based on the c_p^{min} @ 10K case study (see Table 1 in the main article). The main reason we create ∼5% structures that are invalid is due to step (1) above. Specifically, despite the robustness of SELFIES and our optimisation algorithm only retaining valid linkers (see Supplementary note 4), we can fail to embed (use distance geometry to obtain the initial 3D coordinates for a molecule) the linker molecule from its string-based representation using RDKit.[14] This means there are no conformers available for generating the appropriate 3D coordinates in XYZ format, which is needed to construct a CIF file using *pormake*.

- **Uniqueness:** For the 10,000 MOF linkers optimised in the c_p^{min} @ 10K case study, 99.4% of them are unique (as reported in Table 1 of the main article).

Sm-VAE

- **Summary:** *Benefits:* Sm-VAE is capable of unconditional and conditional generation of MOFs, and can optimise the full MOF structure (topology, node, and chemical linker). Also, because of its well-organised latent space, Sm-VAE is capable of interpolating between known structures, or exploring local latent chemical spaces based on distance measures (MOFs that are closer in the latent space have similar features). In principle, global optimisation algorithms can be used to optimise MOFs within the latent space of Sm-VAE, however only local gradient-based optimisation is explored in their work. *Drawbacks:* Sm-VAE has the greatest training requirements of the models considered in Table S6. The structural validity of MOFs sampled is also relatively low at 61.5% because both the reticular information and the linker SELFIES representation is optimised simultaneously.

- **Overview:** Sm-VAE (supramolecular variational autoencoder) by Yao et al.[3] is built on the principles of a VAE, which consists of an encoder and a decoder. The encoder

compresses the input data — in this case, a string representation known as reticular framework (RF) codes, which decomposes the frameworks into chemical linkers (as SELFIES strings), (in)organic nodes, and topologies (as categorical variables) — into a lower-dimensional latent space, and the decoder reconstructs the data from this latent space. Once properly trained, sampling from the learned latent space recovers a distribution of materials that follows the training distribution, i.e., one can unconditionally generate new MOF structures that resemble those used to train the model. As the latent space is a vector space (and is therefore differentiable, similar to deep dreaming's vectorised representation within the real chemical space), it is amicable to continuous optimisation, i.e., one can bias the generative process to produce new MOF structures with targeted properties. To achieve this, one must incorporate some information on the structure-property mapping within the model itself. As such, Yao et al.[3] train Sm-VAE on a large dataset of unlabelled frameworks and a smaller dataset with property labels, optimising the model to predict properties effectively. Property-guided optimisation then occurs within the latent space using a Gaussian process (GP) model, trained on the latent vectors of MOFs and their property values, to search for new MOFs with improved properties.

- **Training requirements:** In order to (unconditionally) generate realistic samples (MOFs), approximately 2 million unlabelled MOF structures were needed to train the model. To incorporate information on the structure-property mappings, 45,000 MOFs were labelled with the relevant property values. Sm-VAE therefore has the highest training requirements of the models considered in Table S6. Both the labelled and unlabelled MOF structures were generated in-house with an augmented linker dataset (of ∼300,000 linkers) and a limited number of nodes / topologies using the ToBaCCo[15] constructor.

- **Design focus:** Because the full RF code is projected to a continuous latent space,

the entire MOF structure (topology code, the category of the nodes, and the SELFIES representation of the linker) can be optimised.

- **Unconditional or targeted generation:** As discussed above, both unconditional and conditional generation can be achieved.

- **Optimisation approach:** A GP predictor model is used to locally optimise MOFs within the latent space of the Sm-VAE model using gradient-based algorithms.

- **Validity:** Yao et al.[3] report that randomly sampling 10,000 points from the trained latent space of Sm-VAE (also referred to as the prior validity) recovers approximately 6,150 valid RF codes (i.e., the correct reticular information paired with valid SMILES strings for the organic linker). The authors also report that, of the valid RF codes generated by unconditional sampling, 99.6% of them are successfully reconstructed by the Sm-VAE model (otherwise referred to as the posterior validity). We take the overall structural validity of MOFs within this framework to simply be the prior validity of 61.5%, given how high the posterior validity is (i.e., we assume the posterior validity to be 100%, and that all MOFs would successfully undergo structural relaxation).

- **Uniqueness:** A uniqueness metric was not reported by Yao et al.[3] However, by running their code (`https://doi.org/10.24433/CO.8185164.v1`) and reproducing the results of their original publication, we are able to recover the raw reticular framework (RF) codes generated from their prior validity checks. 99.7% of the (valid) SMILES strings that encode the MOF linkers are unique, i.e., 99.7% of 6,150 linkers.

GHP-MOFassemble

- **Summary:** *Benefits:* GHP-MOFassemble is capable of generating novel linker chemistries at an atomic level directly in 3D coordinates. The approach also relies on available datasets and models for training: the diffusion model (used for generating linkers) is

trained on standard benchmark datasets (such as ZINC, CASF, and GEOM), and the property predictor is trained on a separate labelled MOF dataset. This is less cumbersome than producing the training data from scratch. *Drawbacks:* The design focus is rather limited, as it only considers 3 different inorganic nodes and a single topology. There is also no capability for targeted generation — the performance of MOFs must be screened separately rather than directly optimised. The validity and uniqueness metrics for this approach are also the lowest out of all models in Table S6.

- **Overview:** GHP-MOFassemble presents a multistep approach towards discovering new MOFs with enhanced CO_2 capture capabilities. The process begins with decomposing high-performing MOF linkers from an existing dataset into molecular fragments. Using the diffusion model DiffLinker,[16] new MOF linkers are generated by bridging these fragments and evaluating them for synthesisability, validity, and uniqueness. Valid linkers are then assembled with pre-selected metal nodes into novel MOF structures. The performance of these MOFs for carbon capture applications are then screened in a separate step using a pre-trained modified Crystal Graph Convolutional Neural Network (CGCNN).

- **Training requirements:** GHP-MOFassemble relies on two separate pre-trained modules for (1) MOF linker generation, and (2) MOF property prediction. MOF linkers are generated using DiffLinker, which is a diffusion model trained on the order of 10^5 organic molecules from datasets such as ZINC (`https://zenodo.org/records/7121271`), CASF (`https://zenodo.org/records/7121264`), and GEOM (`https://zenodo.org/records/7121278`). For MOF property prediction, Park et al.[12] use a pre-trained CGCNN, trained on approximately 137,000 labelled MOF structures from the hMOF dataset of Bobbit et al.,[17] to screen for CO_2 adsorption capacities at 0.1 bar.

- **Design focus:** The main focus is towards generating novel linker chemistries. The

authors do not actively search for optimal MOF nodes or topologies.

- **Unconditional or targeted generation:** As no information on the structure-property mapping is posited into DiffLinker, there is no capability for targeted generation. Only unconditional generation of organic linkers is possible within this approach.

- **Optimisation approach:** There is no direct optimisation of MOFs in this work.

- **Validity:** The value of 15.6% reported in Table S6 is calculated directly from the discussion of Park et al.:[12] the authors constructed 120,000 MOFs by randomly sampling from 12,305 valid linkers generated by the DiffLinker[16] model and combined these linkers with one of three common inorganic node SBUs from the hMOF dataset of Bobbit et al.[17] Of these 120,000 MOFs, only 18,770 MOFs (15.6%) are deemed valid based on: (1) interatomic distance checks, and (2) the number of structures that are amicable to structural relaxation using the UFF4MOF[18,19] forcefield.

- **Uniqueness:** We compute the uniqueness of generated linkers in Table S6 by referring to Table 4 in the original article of Park et al.[12] Specifically, the authors report the fraction of unique linkers amongst various subsets of the full 12,305 linkers generated by DiffLinker. We therefore computed the number of unique linkers within each subset, sum them, and then divide this number by 12,305 to obtain a percentage of 67.5%.

RL transformer

- **Summary:** *Benefits:* RL transformer by Park et al.[13] can optimise full MOF structures using a global optimisation strategy (reinforcement learning). This optimisation also occurs within the real chemical space, which is more interpretable than, for example, the Sm-VAE optimisation strategy. *Drawbacks:* The training requirements are quite similar to Sm-VAE (large-scale self-supervised learning on millions of unlabelled structures, and then fine-tuning using smaller subsets of labelled structures). As the

generator utilises a batch of scaffolds to construct organic linkers, the uniqueness metric is relatively low compared to deep dreaming and Sm-VAE. This more coarse-grained approach also means that linkers are not optimised with atomic resolution.

- **Overview:** The reinforcement learning (RL) transformer approach by Park et al.[13] represents MOF structures similar to Sm-VAE: metal nodes and topologies are encoded as categorical variables, and organic linkers are encoded using SELFIES strings. The framework comprises two main components: (1) a transformer generator (acting as the agent), which creates new MOFs by selecting topologies and metal nodes sequentially, and then generates organic linkers using a scaffold-based approach where up to 4 chemically semantic fragments are joined to create new linker scaffolds. (2) The predictor, functioning as the environment, evaluates these generated structures to estimate their CO_2 capture performance (i.e., CO_2 heat of adsorption at infinite dilution and CO_2/H_2O Henry's selectivity), returning a reward signal based on these properties. This reward is used to iteratively refine the generator's parameters through a policy gradient method, which aims to maximise the expected cumulative reward by improving the quality of the generated MOFs over successive iterations. The training process involves pre-training the generator on a large dataset of MOFs before fine-tuning it with the reinforcement learning algorithm. During this process, a dual-generator approach is employed to balance exploration and exploitation, with one generator focusing on known good strategies (exploitation) and the other exploring new possibilities (exploration).

- **Training requirements:** To embed basic chemical knowledge and patterns, the generator is trained in a self-supervised manner on an unlabelled dataset of approximately 1.9 million MOFs. These MOFs were created using *pormake*, where nodes and topology codes were retained without modification, while an augmented edge dataset of approximately 30,000 linkers was created by merging linkers stored within the *por-*

make software. To generate MOFs with targeted property values, the RL framework is used in conjunction with a property predictor (the environment), which required between 24,000–33,000 labelled MOF structures depending on the property of interest (CO_2 heat of adsorption or Henry's selectivity).

- **Design focus:** The RL policy learns to select optimal topologies, metal nodes, and batches of scaffolds to construct MOFs. Therefore, the full MOF structure is optimised.

- **Unconditional or targeted generation:** The generator (before fine-tuning) learns to create MOF samples unconditionally, while targeted generation occurs via reinforcement learning. Therefore, both conditional and unconditional samples may be drawn.

- **Optimisation approach:** Reinforcement learning is used to generate MOFs with preferred material properties, which is a global optimisation approach. This optimisation also occurs directly on the string-based representation of the MOF, i.e., within the real chemical space.

- **Validity:** For a test of 10,000 MOFs, A validity of 69% is reported by Park et al.,[13] prior to optimising their transformer generator using reinforcement learning (i.e., "scratch" in Table 1 of the original article). This metric is computed on the basis that the transformer generator is able to: (1) produce the correct reticular information (match connection points of metal clusters and organic linkers in a given topology), and (2) produce chemically valid linkers. This validity evaluation is similar to that used for the Sm-VAE model. We also assume here that, of the MOFs considered valid, all of them would undergo structural relaxation successfully. As such, we retain the value of 69% in Table S6 for RL transformer.

- **Uniqueness:** The uniqueness of 79% for generated linkers was also taken directly from the work of Park et al.[12] without modification.

Supplementary Note 10: Comparing linker string representations

We explored the use of other textual representations — such as SMILES[20] and DeepSMILES[21] — to encode the linkers in our MOF strings. In Figure S13, we find that SMILES and DeepSMILES outperform Group SELFIES in the *forward* process. This may be attributed to the context-dependent nature of Group SELFIES tokens, which can represent different chemical entities or structural features depending on their position in the string.[1] While this particular feature is what imbues it with 100% chemical robustness, it likely leads to greater difficulty during learning. Similar challenges have been noted with DeepSMILES compared to SMILES, where the more advanced grammar of DeepSMILES sometimes leads to less effective generative modelling outcomes.[22]

However, our primary focus is on the *reverse* process, where we modify the MOF linker representation directly in the real chemical space using gradient-based algorithms. This works best with an optimisation landscape where every point is chemically valid — a condition that SELFIES, known for its semantic and syntactic robustness, uniquely fulfils. For example, we found that SMILES-based representations significantly underperform in deep dreaming operations due to their tendency to produce semantically and / or syntactically invalid molecular strings (Figure S14). Therefore, SELFIES strings are the preferred choice for our framework, aligning with the demands of the deep dreaming process.

[1] To satisfy syntactic robustness, (Group) SELFIES tokens are overloaded, meaning a token can be used to define a chemical entity or can be used to define the length of a branch, for example. The token '[O]' may be interpreted as an oxygen atom, or it may be interpreted as a branch of length 10, depending on whether it appears directly after a branch or ring token in a SELFIES string. For Group tokens, a similar overloading takes place when interpreting the attachment points of the group: an '[O]' after a Group token is interpreted as a relative index of 6, meaning that subsequent tokens after '[O]' would be generated from the 6th attachment point index relative to the initial Group token attachment point. SELFIES also implements a series of derivation rules to satisfy semantic constraints (i.e., valency) and generate valid molecules. In deriving SMILES molecules from (Group) SELFIES strings, the (Group) SELFIES string is traversed, and each token is interpreted as a rule vector that depends on the state of the derivation. For example, '[=O]' may be translated to a '=O' or 'O' SMILES token (i.e., an oxygen atom which shares two pairs of electrons or one pair of electrons with another atom), depending on the preceding tokens.

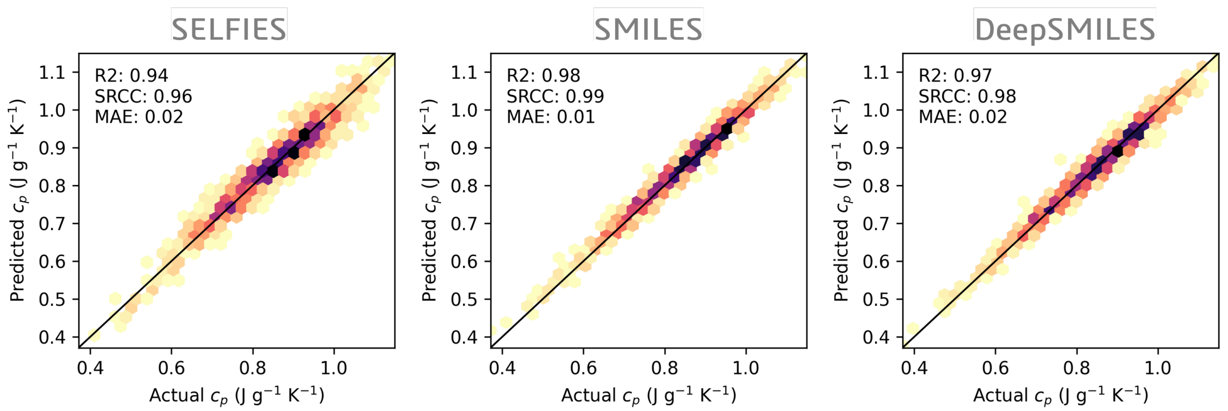

Figure S13: **Parity plots of the deep dreaming model out-of-sample predictive performance for c_p using different string-based representations to encode MOF linkers.** Here we compare ML models built upon strings derived from the Group SELFIES (left), SMILES (middle), and DeepSMILES (right) representations. We use 10,000, 1,000, and 1,000 MOFs for training, validation, and testing purposes in each case study, and apply noise to the zero-elements of the one-hot encodings by sampling from a uniform distribution using k=0.6. Source data are provided as a Source Data file.

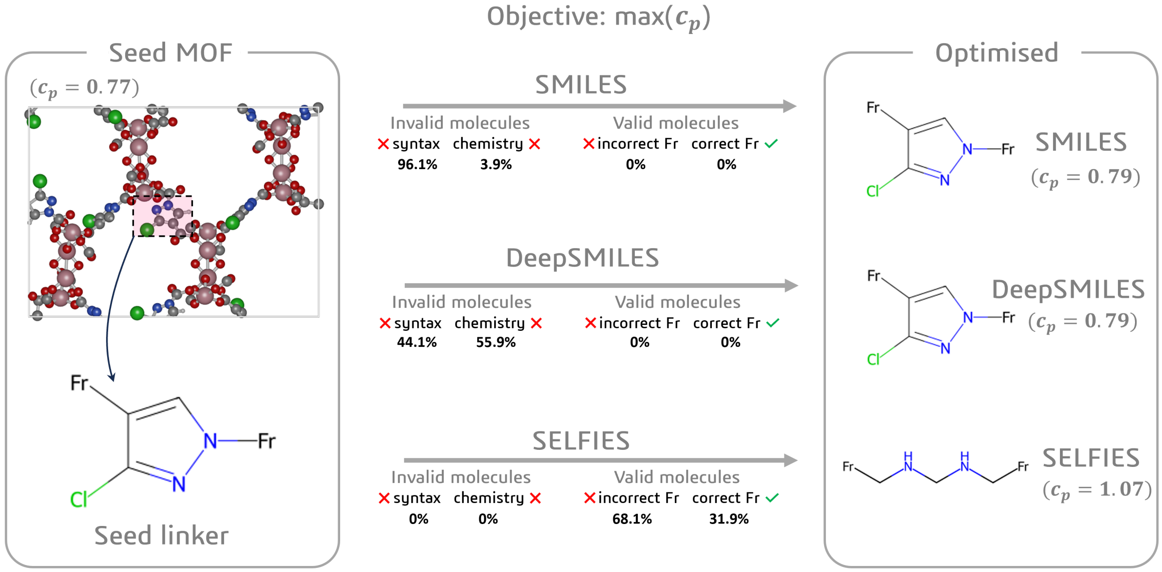

Figure S14: A single deep dreaming case study, whereby we attempt to optimise the c_p of a seed MOF towards higher values, using ML models trained on different molecular string representations to encode the linkers of MOFs. In the case of SMILES, most of the generated strings are syntactically incorrect (violate the SMILES vocabulary), while a smaller number of generated strings (3.9%) are syntactically correct but do not correspond to a chemically valid molecular graph (i.e., are semantically incorrect). DeepSMILES — developed to overcome syntactic limitations of SMILES strings — produce fewer syntactically incorrect strings, but still fail to overcome the semantic limitations within the deep dreaming framework. Group SELFIES strings, on the other hand, only produce valid molecules, 31.9% of which are valid linkers (i.e., have the correct number of connection points, represented as francium pseudoatoms).

References

(1) Cheng, A. H.; Cai, A.; Miret, S.; Malkomes, G.; Phielipp, M.; Aspuru-Guzik, A. Group SELFIES: a robust fragment-based molecular string representation. *Digital Discovery* **2023**, *2*, 748–758.

(2) Krenn, M.; Häse, F.; Nigam, A.; Friederich, P.; Aspuru-Guzik, A. Self-referencing embedded strings (SELFIES): A 100% robust molecular string representation. *Machine Learning: Science and Technology* **2020**, *1*, 045024.

(3) Yao, Z.; Sánchez-Lengeling, B.; Bobbitt, N. S.; Bucior, B. J.; Kumar, S. G. H.; Collins, S. P.; Burns, T.; Woo, T. K.; Farha, O. K.; Snurr, R. Q.; Aspuru-Guzik, A. Inverse design of nanoporous crystalline reticular materials with deep generative models. *Nature Machine Intelligence* **2021**, *3*, 76–86.

(4) Chung, Y. G.; Haldoupis, E.; Bucior, B. J.; Haranczyk, M.; Lee, S.; Zhang, H.; Vogiatzis, K. D.; Milisavljevic, M.; Ling, S.; Camp, J. S.; others Advances, updates, and analytics for the computation-ready, experimental metal–organic framework database: CoRE MOF 2019. *Journal of Chemical & Engineering Data* **2019**, *64*, 5985–5998.

(5) Bovia, D. S. Materials studio. 2019.

(6) Jablonka, K. M.; Ongari, D.; Moosavi, S. M.; Smit, B. Big-Data Science in Porous Materials: Materials Genomics and Machine Learning. *Chemical Reviews* **2020**, *120*, 8066–8129.

(7) Moosavi, S. M.; Novotny, B. Á.; Ongari, D.; Moubarak, E.; Asgari, M.; Kadioglu, Ö.; Charalambous, C.; Ortega-Guerrero, A.; Farmahini, A. H.; Sarkisov, L.; others A data-science approach to predict the heat capacity of nanoporous materials. *Nature materials* **2022**, *21*, 1419–1425.

(8) Lee, S.; Kim, B.; Cho, H.; Lee, H.; Lee, S. Y.; Cho, E. S.; Kim, J. Computational Screening of Trillions of Metal–Organic Frameworks for High-Performance Methane Storage. *ACS Applied Materials & Interfaces* **2021**, *13*, 23647–23654.

(9) Cipcigan, F.; Booth, J.; Ferreira, R. N. B.; dos Santos, C. R.; Steiner, M. Discovery of novel reticular materials for carbon dioxide capture using GFlowNets. *Digital Discovery* **2024**, *3*, 449–455.

(10) Fu, X.; Xie, T.; Rosen, A. S.; Jaakkola, T.; Smith, J. MOFDiff: Coarse-grained Diffusion for Metal-Organic Framework Design. *arXiv preprint arXiv:2310.10732* **2023**,

(11) Park, J.; Lee, Y.; Kim, J. Multi-modal conditioning for metal-organic frameworks generation using 3D modeling techniques. *ChemRXiv* **2024**,

(12) Park, H.; Yan, X.; Zhu, R.; Huerta, E. A.; Chaudhuri, S.; Cooper, D.; Foster, I.; Tajkhorshid, E. A generative artificial intelligence framework based on a molecular diffusion model for the design of metal-organic frameworks for carbon capture. *Communications Chemistry* **2024**, *7*, 21.

(13) Park, H.; Majumdar, S.; Zhang, X.; Kim, J.; Smit, B. Inverse design of metal–organic frameworks for direct air capture of CO2 via deep reinforcement learning. *Digital Discovery* **2024**, *3*, 728–741.

(14) RDKit: Open-source cheminformatics. `https://www.rdkit.org`, Accessed: 2024-02-08.

(15) Colón, Y. J.; Gómez-Gualdrón, D. A.; Snurr, R. Q. Topologically Guided, Automated Construction of Metal–Organic Frameworks and Their Evaluation for Energy-Related Applications. *Crystal Growth & Design* **2017**, *17*, 5801–5810.

(16) Igashov, I.; Stärk, H.; Vignac, C.; Schneuing, A.; Satorras, V. G.; Frossard, P.;

Welling, M.; Bronstein, M.; Correia, B. Equivariant 3D-conditional diffusion model for molecular linker design. *Nature Machine Intelligence* **2024**, 1–11.

(17) Bobbitt, N. S.; Shi, K.; Bucior, B. J.; Chen, H.; Tracy-Amoroso, N.; Li, Z.; Sun, Y.; Merlin, J. H.; Siepmann, J. I.; Siderius, D. W.; others MOFX-DB: An online database of computational adsorption data for nanoporous materials. *Journal of Chemical & Engineering Data* **2023**, *68*, 483–498.

(18) Addicoat, M. A.; Vankova, N.; Akter, I. F.; Heine, T. Extension of the Universal Force Field to Metal–Organic Frameworks. *Journal of Chemical Theory and Computation* **2014**, *10*, 880–891.

(19) Coupry, D. E.; Addicoat, M. A.; Heine, T. Extension of the Universal Force Field for Metal–Organic Frameworks. *Journal of Chemical Theory and Computation* **2016**, *12*, 5215–5225.

(20) Weininger, D. SMILES, a chemical language and information system. 1. Introduction to methodology and encoding rules. *Journal of chemical information and computer sciences* **1988**, *28*, 31–36.

(21) O'Boyle, N.; Dalke, A. DeepSMILES: an adaptation of SMILES for use in machine-learning of chemical structures. *ChemRxiv preprint 10.26434/chemrxiv.7097960.v1* **2018**,

(22) Arús-Pous, J.; Johansson, S. V.; Prykhodko, O.; Bjerrum, E. J.; Tyrchan, C.; Reymond, J.-L.; Chen, H.; Engkvist, O. Randomized SMILES strings improve the quality of molecular generative models. *Journal of cheminformatics* **2019**, *11*, 1–13.
