# Supplementary material for: Inverse design of metal-organic frameworks using deep dreaming approaches
Source: Nat Commun. 2025 May 23;16:4806. doi: 10.1038/s41467-025-59952-3 (PMC12102185; doi:10.1038/s41467-025-59952-3)
Supplement: Supplementary file 2 — Reporting Summary [file 41467_2025_59952_MOESM2_ESM.pdf]

nature portfolio

Corresponding author(s): Conor Cleeton

Last updated by author(s): 26/03/2025

Reporting Summary

Nature Portfolio wishes to improve the reproducibility of the work that we publish. This form provides structure for consistency and transparency in reporting. For further information on Nature Portfolio policies, see our Editorial Policies and the Editorial Policy Checklist.

Statistics

For all statistical analyses, confirm that the following items are present in the figure legend, table legend, main text, or Methods section.

n/a	Confirmed	
☐	☒	The exact sample size (*n*) for each experimental group/condition, given as a discrete number and unit of measurement
☐	☒	A statement on whether measurements were taken from distinct samples or whether the same sample was measured repeatedly
☐	☒	The statistical test(s) used AND whether they are one- or two-sided<br>*Only common tests should be described solely by name; describe more complex techniques in the Methods section.*
☒	☐	A description of all covariates tested
☐	☒	A description of any assumptions or corrections, such as tests of normality and adjustment for multiple comparisons
☐	☒	A full description of the statistical parameters including central tendency (e.g. means) or other basic estimates (e.g. regression coefficient) AND variation (e.g. standard deviation) or associated estimates of uncertainty (e.g. confidence intervals)
☒	☐	For null hypothesis testing, the test statistic (e.g. *F*, *t*, *r*) with confidence intervals, effect sizes, degrees of freedom and *P* value noted<br>*Give P values as exact values whenever suitable.*
☒	☐	For Bayesian analysis, information on the choice of priors and Markov chain Monte Carlo settings
☒	☐	For hierarchical and complex designs, identification of the appropriate level for tests and full reporting of outcomes
☐	☒	Estimates of effect sizes (e.g. Cohen's *d*, Pearson's *r*), indicating how they were calculated

Our web collection on statistics for biologists contains articles on many of the points above.

Software and code

Policy information about availability of computer code

Data collection	Hypothetical MOF structures generated using PORMAKE. Source data and scripts are available at https://github.com/SarkisovTeam/dreaming4MOFs
Data analysis	RASPA2 (adsorption simulations, as implemented in MOFDSCRIBE package v.0.0.9.dev0), zeo++ (structural analysis, v.0.3), specific heat capacity (machine learning model, accessible via https://github.com/SeyedMohamadMoosavi/tools-cp-porousmat) and bandgap (CGCNN machine learning model accessible via https://github.com/txie-93/cgcnn). Source data and scripts are available at https://github.com/SarkisovTeam/dreaming4MOFs

For manuscripts utilizing custom algorithms or software that are central to the research but not yet described in published literature, software must be made available to editors and reviewers. We strongly encourage code deposition in a community repository (e.g. GitHub). See the Nature Portfolio guidelines for submitting code & software for further information.

Data

Policy information about availability of data

All manuscripts must include a data availability statement. This statement should provide the following information, where applicable:

- Accession codes, unique identifiers, or web links for publicly available datasets
- A description of any restrictions on data availability
- For clinical datasets or third party data, please ensure that the statement adheres to our policy

The source data (scripts, training data, pre-trained models, CIF files, dreaming results) and code are freely available at https://github.com/SarkisovTeam/dreaming4MOFs

Research involving human participants, their data, or biological material

Policy information about studies with human participants or human data. See also policy information about sex, gender (identity/presentation), and sexual orientation and race, ethnicity and racism.

Reporting on sex and gender	n/a
Reporting on race, ethnicity, or other socially relevant groupings	n/a
Population characteristics	n/a
Recruitment	n/a
Ethics oversight	n/a

Note that full information on the approval of the study protocol must also be provided in the manuscript.

Field-specific reporting

Please select the one below that is the best fit for your research. If you are not sure, read the appropriate sections before making your selection.

☐ Life sciences ☐ Behavioural & social sciences ☒ Ecological, evolutionary & environmental sciences

For a reference copy of the document with all sections, see nature.com/documents/nr-reporting-summary-flat.pdf

Ecological, evolutionary & environmental sciences study design

All studies must disclose on these points even when the disclosure is negative.

Study description	Optimising the linkers of MOFs using "deep dreaming", where a regression model - trained on textual representations - is inverted to design new MOFs with improved functionalities.
Research sample	hypothetical metal-organic frameworks
Sampling strategy	12,000 hypothetical MOFs were randomly generated to train our machine learning models.
Data collection	Samples were generated using PORMAKE, a hypothetical MOF constructor
Timing and spatial scale	n/a
Data exclusions	MOFs with unit cell lengths greater than 60 Angstroms, or whose number of atoms in the unit cell are greater than 1,500, were excluded from our training datasets.
Reproducibility	Jupyter notebooks are provided to reproduce the results
Randomization	A random split of 10,000, 1,000, and 1,000 MOFs were used for training / validation / testing purposes to train the machine learning models.
Blinding	n/a

Did the study involve field work? ☐ Yes ☒ No

Reporting for specific materials, systems and methods

We require information from authors about some types of materials, experimental systems and methods used in many studies. Here, indicate whether each material, system or method listed is relevant to your study. If you are not sure if a list item applies to your research, read the appropriate section before selecting a response.

Materials & experimental systems

n/a	Involved in the study
☒	☐ Antibodies
☒	☐ Eukaryotic cell lines
☒	☐ Palaeontology and archaeology
☒	☐ Animals and other organisms
☒	☐ Clinical data
☒	☐ Dual use research of concern
☒	☐ Plants

Methods

n/a	Involved in the study
☒	☐ ChIP-seq
☒	☐ Flow cytometry
☒	☐ MRI-based neuroimaging

Plants

Seed stocks	n/a
Novel plant genotypes	n/a
Authentication	n/a
